# 

**Published:** 1905-06-10

**Authors:** 


### NOTICE TO CORRESPONDENTS.

All MSS., Letters, Books for Review, and other matters intended for the Editor should be addressed The Editor, "The Hospital," The Hospital Buildings, 28 & 29 Southampton Street, Strand, W.C.

The Editor cannot undertake to return rejected MS., even when accompanied by stamped directed envelope.

**Notice to Advertisers and Subscribers.**

All Advertisements, Orders for Copies of The Hospital, and Business Communications should be addressed to **THE MANAGER (Not to the Editor),** The Hospital, 28 & 29 Southampton Street, Strand, London, W.C.

The Cost of Subscription, post free, is at follows:—Per week, 3½d.; per uarter, 3s. 3d.; per half-year, 6s. 6d.; per annum, 13s. Foreign and olonial:—Per annum, 19s. 6d., payable, in all cases, in advance.

**NOTICE.—Vol. XXXVII. of "The Hospital" October 1904 to March 1905), in handsome cloth binding, will shortly be on sale at the office of this Journal, price 10s. 6d. Cases for binding the Numbers contained in this Volume 2s. Index to Vol. XXXVII., 3½d., post free.**

**The Monthly Part for June is now ready. Price 1s., by post, 1s. 3d.**

### BOOKS RECEIVED.

H. J. Glaisher.

"Muco-membranous Enterocolitis." By Paul Froussard, M.D.

A. H. Goose, Norwich.

"The Ritual of Temperance and State Hygiene." By H. Cooper Patten, M.D.

J. Wright and Co., Bristol.

"Golden Rules of Medical Practice." By Lewis Smith, M.D.

The Scientific Press, Limited.

"A Handbook for Nurses." Third Edition. By J. K. Watson, M.D.

"How to Become a Nurse: The Nursing Profession, How and Where to Train." By Sir Henry Burdett.

"Hints to Nurses on Tropical Fevers." By S. F. Pollard.

### Medical Appointments.

**George Ashton**, M.D.Manch., M.R.C.S.Eng., L.R.C.P.Lond., Medical Officer for Home Patients to the Manchester Royal Infirmary. **D. A. Beattie**, M.D.Toronto, Clinical Assistant to the Chelsea Hospital for Women. **Thomas Creaser**, B.A., M.B., Ch.B.Dub., Junior House Surgeon to the Bootle Borough Hospital. **John Dodd**, M.D., M.Ch. R.U.I., Visiting Medical Officer to the Leicester Poor-law Infirmary. **Spencer Smithson Dunn**, M.B., Ch.M.Aberd., Vaccinator for the District of Victoria, Australia. **C. P. W. Dyring**, M.B.Melb., Certifying Medical Practitioner for the purposes of the Factories and Shops Acts, Victoria, Australia. **L. E. Ellis**, M.B., Ch.M.Syd., Medical Officer of Manilla Hospital, New South Wales, Australia. **Walker Fisher**, M.R.C.P.Edin., L.F.P.&S.Glasg., Government Medical Officer at Weston, Maitland, and Hon. Visiting Officer to the Maitland Hospital, New South Wales, Australia. **Alexander Young Fullerton**, L.R.C.P.Lond., M.R.C.S.Eng., Licensing Magistrate and Official Member of the Licensing Court for the Licensing District of Murrurundi, New South Wales, Australia. **E. A. Graham**, M.B., Ch.B.Melb., Certifying Medical Practitioner for the purposes of the Factories and Shops Acts, Victoria, Australia. **Stanley Hodgson**, M.D., B.S.Lond., M.R.C.S.Eng., Honorary Physician to the Pendleton Branch Dispensary of the Royal Salford Hospital. **Cyril A. B. Horsford**, M.D.Edin., F.R.C.S.Eng., reappointed Senior Clinical Assistant to the Throat Hospital, Golden Square. **J. Howard-Jones**, M.R.C.S.Eng., L.R.C.P.Lond., Medical Officer and Public Vaccinator to the Worthen District and Medical Officer of Health to the Chirbury Rural District, Shropshire. **William Ernest Jones**, M.R.C.S. Eng., L.R.C.P.Lond., Inspector-General of the Insane, Victoria, Australia. **Walter Edward Manby**, B.A., M.B., B.Ch.Cantab., L.R.C.P. Lond., M.R.C.S., Medical Officer of Health of Bridport, Dorset. **Wright Mitchell**, B.A., M.B., Ch.B.Dub., Senior House Surgeon to the Bootle Borough Hospital. **W. Salisbury Sharpe**, M.D., M.R.C.S., M.R.C.P., Clinical Assistant to the Chelsea Hospital for Women. **George Nicol Wilson**, M.D., D.P.H. Aberd., Senior Assistant Medical Officer to the Port of Glasgow.

Just Published.
Price 2/4 post free.

New and
Revised Edition.

### HOW TO BECOME A NURSE.

### THE NURSING PROFESSION: How and Where to Train.

Edited by Sir HENRY BURDETT, K.C.B.

This work is an indispensable guide to those desiring to enter the Nursing Profession.

### THE BURDETT SERIES OF POPULAR NURSING TEXT-BOOKS.

The Volumes which comprise this Series are written by a competent authority on the subject treated with the object of making each work of the utmost practical value to Nurses. Each Volume contains about 100 pages, well bound in cloth boards.

No. 1.—**PRACTICAL HINTS ON DISTRICT NURSING.** By Miss Amy Hughes.

No. 2.—**HOSPITAL AND PRIVATE NURSING.** By Miss S. E. Orme.

No. 3.—**THE MIDWIVES' POCKET-BOOK** (with Prefatory Chapter on the Midwives Bill). By Miss Honnor Morten.

No. 4.—**FEVERS & INFECTIOUS DISEASES:** their Nursing and Practical Management. By William Harding, M.D.Ed., M.R.C.P.Lond.

No. 5.—**NOTES ON PHARMACY AND DISPENSING FOR NURSES.** By C. J. S. Thompson.

No. 8.—**HOME NURSING OF SICK CHILDREN.** By J. D. E. Mortimer, M.B., F.R.C.S.

No. 9.—**MENTAL NURSING.** By William Harding, M.B. Ed., M.R.C.P. Lond.

No. 10.—**SICK NURSING AT HOME.** By Miss L. G. Moberly.

No. 11.—**GYNÆCOLOGICAL NURSING.** By G. A. Hawkins-Ambler, F.R.C.S.

No. 12.—**SYLLABUS OF LECTURES TO NURSES.** By Andrew Davidson, M.D.

**Any of the above Books can be obtained separately, price 1/- each post free.**

*PUBLISHED BY*

**The Scientific Press, Ltd.,** Publishers of Nursing Text-Books, Case-Books & Charts of all descriptions,

28 & 29 SOUTHAMPTON STREET, STRAND, LONDON, W.C.

*Write for New Catalogue of Nursing Manuals.*

### The NURSING MIRROR.

**The Nurse's Own Paper.**

**Price ONE PENNY Weekly.**

Of all Booksellers and Newsagents, or posted direct from the Office.

*Write for Subscription Rates to The Manager,*

THE NURSING MIRROR, The Hospital Building, 28 & 29 Southampton Street, Strand, London, W.C.

